# The role of dimensionality in neuronal network dynamics

**DOI:** 10.1038/srep29640

**Published:** 2016-07-11

**Authors:** Francesco Paolo Ulloa Severino, Jelena Ban, Qin Song, Mingliang Tang, Ginestra Bianconi, Guosheng Cheng, Vincent Torre

**Affiliations:** 1 Neurobiology Sector, International School for Advanced Studies (SISSA), via Bonomea, 265, 34136 Trieste, Italy; 2 Key Laboratory of Nano-Bio Interface, Suzhou Institute of Nano-tech and Nano-bionics, Chinese Academy of Sciences, 398 Ruoshui Road, Jiangsu 215123, China; 3 Institute of Life Sciences, Southeast University, Sipailou 2, Nanjing 210096, China; 4 School of Mathematical Sciences, Queen Mary University of London, Mile End Rd, London E1 4NS, United Kingdom

## Abstract

Recent results from network theory show that complexity affects several dynamical properties of networks that favor synchronization. Here we show that synchronization in 2D and 3D neuronal networks is significantly different. Using dissociated hippocampal neurons we compared properties of cultures grown on a flat 2D substrates with those formed on 3D graphene foam scaffolds. Both 2D and 3D cultures had comparable glia to neuron ratio and the percentage of GABAergic inhibitory neurons. 3D cultures because of their dimension have many connections among distant neurons leading to small-world networks and their characteristic dynamics. After one week, calcium imaging revealed moderately synchronous activity in 2D networks, but the degree of synchrony of 3D networks was higher and had two regimes: a highly synchronized (HS) and a moderately synchronized (MS) regime. The HS regime was never observed in 2D networks. During the MS regime, neuronal assemblies in synchrony changed with time as observed in mammalian brains. After two weeks, the degree of synchrony in 3D networks decreased, as observed *in vivo*. These results show that dimensionality determines properties of neuronal networks and that several features of brain dynamics are a consequence of its 3D topology.

Neuronal networks in the brain have connections extending in all 3 dimensions (3D), a characteristic that is lost in planar neuronal cultures grown on 2D supports^1^,^2^,^3^: these 2D networks exhibit cell-cell contacts that differ from the complex 3D interactions that occur *in vivo*. Several properties of brain dynamics have been identified and two of them are particularly relevant here: firstly, the coexistence of segregated and global processing, in which specific computations are carried out locally while information and signals are transmitted throughout the entire brain^4^; secondly, the existence of neuronal assemblies which change their degree of correlated activity both in time and in space, generating a variety of rhythms^5^. These basic properties^6^,^7^,^8^,^9^,^10^ could be a consequence of the fact that neuronal networks in the brain are embedded in a 3D space. Indeed, dynamical properties of 3D brain networks could be significantly different from those of 2D cultures.

The understanding of the different dynamical properties of 2D and 3D neuronal networks is relevant not only for basic neuroscience but it is also important for the repair of the nervous system, especially the brain, and for the realization of what is referred to as the “organic electrode” used for chronic implants^11^,^12^. A first step towards this goal is the development of *in vitro* networks of neurons and/or neuronal stem cells grown in appropriate 3D supporting scaffolds. Graphene is a highly conductive hydrophobic material^13^, therefore graphene scaffolds have been used to grow and to electrically stimulate neuronal networks^14^. Moreover, graphene promotes neurite outgrowth^15^,^16^ and reduces the inflammatory response^17^. A second important step for the repair of the nervous system requires that the cultured 3D neuronal networks have physiological and dynamical properties -as close as possible- to those observed in the brain, allowing the transplantation of these 3D networks into the central nervous system.

The present manuscript has two main objectives: firstly, to understand the role of dimensionality in determining the dynamical properties of neuronal networks, and secondly, to make progress towards the repair of lesions in the nervous system. Therefore, we used 3D graphene foam (3D-GF) scaffolds to grow 3D networks of dissociated rat hippocampal neurons, and we compared the properties of 2D and 3D neuronal networks cultured on 2D Glass coverslip (2D Glass), 2D graphene films (2D G), and 3D-GFs. We show that 3D networks have dynamical properties that are quantifiably more similar to what is observed in the brain than 2D networks^4^,^5^,^18^,^19^,^20^.

## Results

Important features of networks depend on their connectivity, i.e. the number and properties of the connections between the units (neurons) composing the networks^21^,^22^.

In order to determine differences between 2D and 3D neuronal networks, we plated hippocampal neurons on different substrates: 2D Glass, 2D G and 3D-GFs and examined their morphology and dynamics after 8–9 and 14–15 days *in vitro* (DIV) using immunocytochemistry and calcium imaging.

### Dynamical properties of 2D and 3D networks

Mechanisms leading to the synchronization of coupled oscillators have been extensively studied for several decades and more recently using network theory^23^,^24^,^25^,^26^,^27^. Novel insights into the global dynamics of coupled oscillators on lattices, where coupling is restricted to the nearest neighbours, clarify the effect of dimensionality on the synchronization properties of these networks. In fact, using tools from mean-field analysis, scaling theory and numerical simulations^28^,^29^ it is possible to investigate the role of the dimension *d* in network formed by the classical Kuramoto model. Indeed, fully entrained synchrony in an infinite hypercubic lattices is possible only for *d* > 4, whereas entrained states can form only locally for 2 < *d* ≤ 4. In addition, synchronization has been shown to be impossible for *d* ≤ 2, i.e., global or local entrained states cannot occur for these dimensions^29^. These results provide a theoretical framework to explain why in 2D lattice no synchronization can occur while in 3D lattices some sort of weak synchronization is expected. Recent developments in network theory^23^,^24^,^25^ have elucidated the role of long-range shortcuts, i.e., of a direct coupling between nodes (neurons) that are not physically near. The modularity of the network, i.e., the tendency of some units to be more densely connected to each other than to the rest of the network, together with the presence of short- and long-range connections (the small-world property) produce a rich phenomenology, including full synchronization and patches of synchronization that vary in time and space, such as frustrated synchronization^9^,^10^.

**Figure 1:**
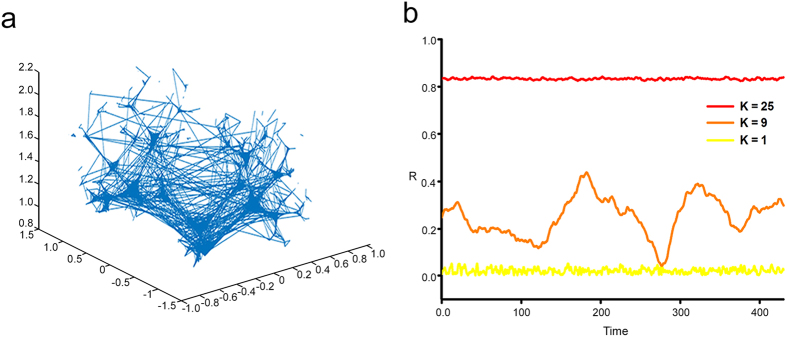
3D network model. (**a**) Simulation of a 3D neuronal network which is modular and has short range connections and some long range connections (small-word network). The neurons are distributed along a fractal tree and primarily connected by short-range interactions; long-range interactions constitute a small proportion of the connections. The Kuramoto model of this network yields three dynamical regimes as a function of the strength of the coupling, K, between the oscillators. Large values of K result in a fully synchronized phase, whereas low values of K do not produce synchronization. Intermediate values of K produce a phase of frustrated synchronization. In panel (**b**), we plotted the order parameter, R, for the synchronization as a function of time, t, for different values of the coupling, K. The parameter R ranges from one (totally synchronized state) to zero (absence of synchronization). As a function of K, the plots indicate three different synchronization phases for the simulated 3D network.

We have developed models in which the 3D spatial distribution of neurons promotes the establishment of neuronal networks with small-world properties and high modularity. To this end we have modelled the 3D scaffold as a fractal tree, and we have sprinkled neurons on it uniformly, generating regions of different neuronal density. Then short distance links and a small density of long distance links were established forming a modular small-world network (Fig. 1a). The obtained networks exhibit three kinds of dynamical regimes (Fig. 1b) depending on the strength of coupling K: full synchronization (red line) was observed for high values of K, whereas low values of K resulted in the absence of synchronization (yellow); intermediate values of K yielded a time-varying degree of synchronization, usually referred as frustrated synchronization (orange). Frustrated synchronization is a consequence of the modularity of the network and of an intermediate value of K. All these results suggest a more complex dynamics in 3D than in 2D neuronal networks.

### 2D and 3D cultures grown on graphene substrates

**Figure 2:**
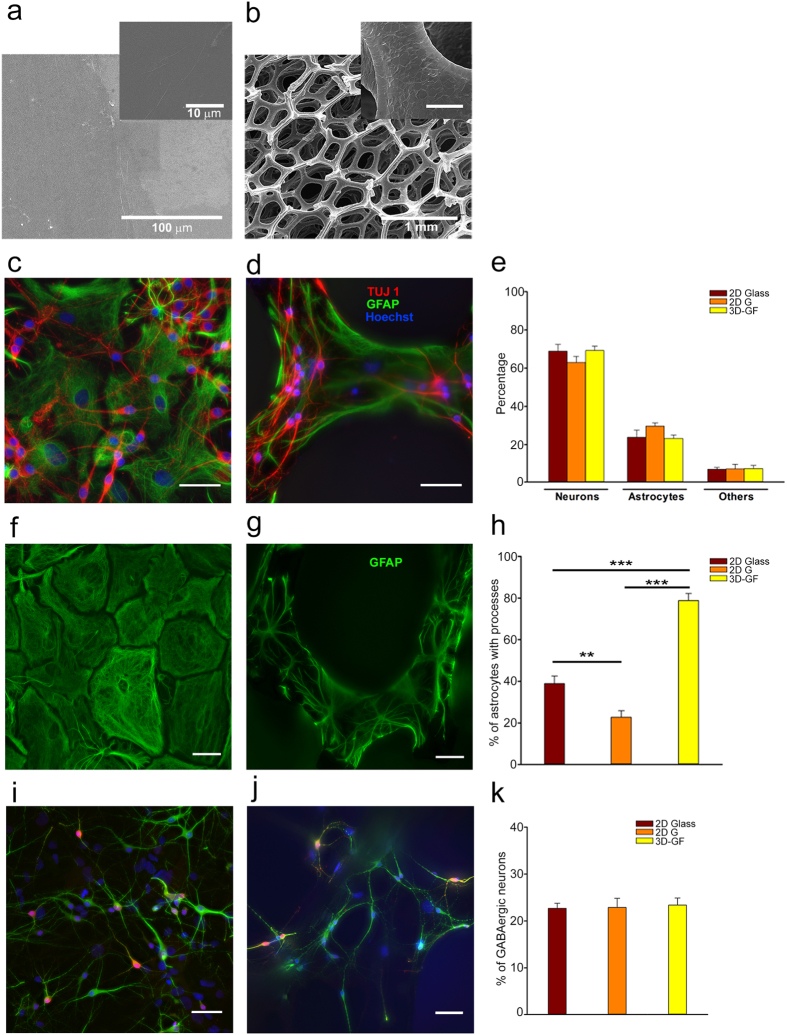
Cellular morphology of 2D and 3D cultures. (**a**) SEM image of a 2D graphene film. Darker areas present an higher number of layers compared to the brighter one; as shown in the inset the surface is flat. (**b**) SEM image of a 3D Graphene Foam scaffold (3D-GF); the surface presents ripples, as shown in the inset. (**c**) Hippocampal culture at 7 DIV on 2D Glass stained for β-tubulin III (TUJ1, red), glial fibrillary acidic protein (GFAP, green) and Hoechst 33342 nuclear stain (blue). (**d**) The same as (**c**) but for 3D-GFs. (**e**) Proportion of neurons (TUJ 1-positive) and glia (GFAP-positive) among different substrates tested. TUJ 1- and GFAP-negative cells are referred to as “other”. (**f,g**) GFAP staining of astrocytes on 2D G and 3D-GF, respectively. (**h**) Percentage of astrocytes with processes for the different substrates tested (**p < 0.01 ***p < 0.001 One-way ANOVA, Holm-Sidak post-hoc test). (**i,j**) Neuronal cultures on 2D G and and 3D-GFs respectively. Cells were stained for MAP2 (green), GABA (red) and Hoechst 33342 nuclear marker (blue). (**k**) Percentage of GABAergic inhibitory neurons for three conditions tested. The images are the projections of a 20 μm z-stack for 2D samples and 35–50 μm for the 3D cultures, acquired with 0.5-μm slice spacing. Scale bar, 50 μm.

2D G and 3D graphene foams (Fig. 2a,b) were prepared using a chemical vapour deposition method using a Cu and Ni template respectively. Successive washing steps (Methods) then chemically removed the templates. Raman spectrum analysis obtained from these 3D-GFs shows that these scaffolds are of high quality and consist of few graphene sheets^14^. 3D-GFs have holes with diameters ranging from 100 to 500 μm and the size of their backbone varied from 100 to 200 μm. Neurons survived well in all three tested conditions and formed functional networks after one week.

In order to examine the relative abundance of neurons and glial cells, cultures were stained with antibodies for neurons (β-tubulin III) and for glial cells with glial fibrillary acidic protein (GFAP), as shown in Fig. 2c,d. The proportion of β-tubulin III-positive neurons (68.89 ± 3.59% on 2D Glass, 63.01 ± 3.18% on 2D G and 69.29 ± 2.23% on 3D-GFs) and the percentage of GFAP-positive astrocytes (23.93 ± 3.66%, 29.67 ± 1.68% and 23.25 ± 1.84% on 2D Glass, 2D G and 3D-GFs, respectively) were similar among different cultures (Fig. 2e; n = 6(744), n = 5(436), n = 7(403)). Glial cells adhered to the 3D-GFs as well as to flat surfaces, forming a layer above which neurons formed a network. Both stellate-shaped and flattened ovoid morphologies were observed (Fig. 2f,g), which are typical of *in vitro* dissociated primary cultures^30^,^31^. Nevertheless, the majority of astrocytes on 3D-GFs extended processes (78.80 ± 3.37%, n = 8 (125)), whereas the ratio of astrocytes with extended processes was significantly lower for 2D cultures (Fig. 2h, 38.82 ± 3.69%, n = 9(432) for 2D Glass and 22.67 ± 3.2%, n = 5(160) for 2D G, one-way ANOVA tested with Holm Sidak test). These observations suggest that 3D cultures favour a more differentiated and *in vivo*-like morphology.

We compared the amount of inhibitory GABAergic neurons between 2D and 3D cultures after 7DIV with staining for microtubule-associated protein 2 (MAP2) and GABA (Fig. 2i,j). As shown in Fig. 2k, all cultures tested showed comparable percentages of GABA-positive neurons: 22.77 ± 1.07% for 2D Glass, 22.90 ± 1.92% for 2D G and 23.40 ± 1.49% for 3D-GFs (n = 5(566), n = 7(436) and n = 11(276) for glass, 2D G and 3D-GFs respectively). These data show that the ratio of excitatory and inhibitory neurons was not altered on 3D-GFs, but the composition of GABAergic subtypes^32^,^33^ or the density of both pre- and post-synaptic GABA receptors could be different.

Neuronal culture on 3D-GFs extended continuously and uniformly along the scaffold backbone (Supplementary Fig. S1). After one week of culture, we counted the number of neurites emerging from each neuron and the average number of neurites was similar for glass, 2D G and 3D-GFs (2.99 ± 0.08, 3.06 ± 0.08 and 2.86 ± 0.08 neurites per neuron; n = 141, 149 and 140 neurons analysed for 2D Glass, 2D G and 3D-GFs respectively). The same analysis showed that frequency distribution of the neurites’ number was similar for the three different supports (Supplementary Fig. S2). After two weeks of culture, axons approached a millimetre in length in both 2D and 3D cultures (Supplementary Fig. S3), but they only vertically extended several hundreds of micrometres on 3D-GFs, allowing a more extensive connectivity (Supplementary Video S1).

### The spontaneous electrical activity of 3D networks is more synchronous

**Figure 3:**
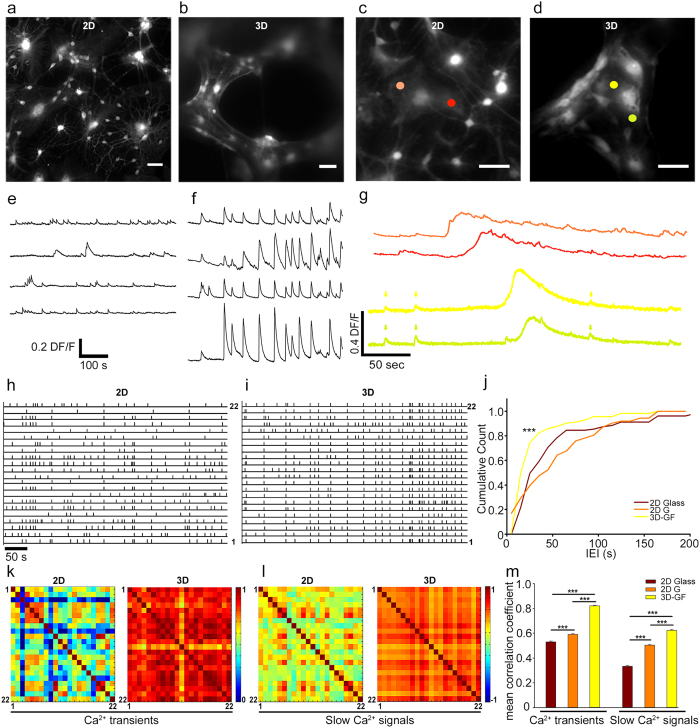
The spontaneous activity of 3D networks is more synchronous. (**a,b**) Neuronal cultures loaded with 4 μM Fluo-4-AM calcium indicator on 2D Glass and 3D-GF, respectively. (**c,d**) Glial cells on 2D G and 3D-GF respectively. Coloured circles indicate the ROIs where calcium waves were obtained. (**e,f**) Calcium transients on 2D Glass and 3D-GF, respectively, for 4 selected neurons. (**g**) Calcium waves from glial cells on 2D G (top traces) and 3D-GFs (bottom traces); arrows indicate signals from neurites projecting over glial cells. (**h**) Raster plot of 2D culture on glass and (**i**) 3D culture on 3D-GFs for 22 selected traces. (**j**) Cumulative count of the Inter Events Interval (IEI) for neuronal cultures grown on 2D Glass, 2D G and 3D-GF (**p < 0.01 ***p < 0.001 ANOVA on ranks, Domm’s post-hoc test). (**k**) Cross-correlation matrices of calcium transients 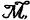 in 2D and 3D neuronal networks. (**l**) Cross-correlation matrices of slow calcium signals 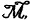 for the same two conditions. (**m**) Mean correlation coefficient of the calcium transients and slow calcium signals (***p < 0.001 ANOVA on ranks, Domm’s post-hoc test). Scale bar, 50 μm.

Fluorescence images of the Fluo-4-loaded neuronal cultures confirmed the formation of 2D neuronal networks (Fig. 3a) plated either on 2D Glass or on 2D G. Fluorescence images of 3D neuronal networks grown on 3D-GFs (Fig. 3b) showed that the scaffold backbone was entirely covered by neurons and glial cells. Cells could be visualized up to 500 μm deep in the scaffold because of the large size of the scaffold pores. Glial cells were present in all cultures and could be morphologically identified both in 2D and 3D cultures (Fig. 3c,d), because of their larger cell body and the shape of their processes. The spontaneous electrical activity of neurons was monitored by measuring their calcium transients (DF/F), obtained by acquiring fluorescence images at 3–10 Hz for 10–20 min. During this recording time, the emitted fluorescence was stable with negligible bleaching (Supplementary Video S2).

After one week of culture, it was possible to record clear calcium transients from both 2D and 3D neuronal cultures (Fig. 3e,f). The onset time of calcium transients was defined by detecting those events in the fluorescence signal that exceed at least three times the standard deviation of the noise (≈0.01 DF/F). Standard analysis of the percentage of active neurons, the mean amplitude of Ca^2+^-transients and the frequency distribution of the amplitude, reveal that neuronal activity is significantly different for three-dimensional cultures (Supplementary Fig. S4). Indeed, calcium transients from 2D and 3D cultures had a different degree of synchrony, with 3D cultures appearing consistently more synchronous. Calcium waves from glial cells (Fig. 3g) were less frequent but longer and larger with amplitude approximately greater than 0.2 DF/F and propagated at a speed between 4 and 5 μm/s in 2D cultures and between 5 and 7 μm/s in 3D-GFs. By contrast, calcium transients in neurons appeared to propagate along neurites almost instantaneously. Superimposed smaller and rapidly propagating calcium transients were observed in calcium waves, these transients presumably originated from neurites grown over glial cells (Fig. 3g arrows). Calcium waves in glial cells propagated both inter- and intra-cellularly along glial processes^34^,^35^.

Raster plots of detected calcium transients from 2D (Fig. 3h) and 3D neuronal networks (Fig. 3i) were constructed. The Δt interval between two successive calcium transients was computed to obtain an average inter events interval (IEI) for each neuron. The cumulative count (Fig. 3j) showed that the IEI for neurons in 3D-GFs was 32.8 ± 3.1 s, whereas this value was 51.5 ± 5.4 s and 57.3 ± 4.4 s for 2D Glass and 2D G, respectively. Therefore, the rate of firing was significantly higher in 3D networks compared to both the 2D cultures (ANOVA on ranks tested with Domm’s post-hoc test. n = 114 neurons for 6 neuronal cultures on 3D-GF; n = 103 neurons for 6 neuronal cultures on 2D Glass; n = 127 neurons for 5 neuronal cultures on 2D G).

On the basis of these raster plots, we analyzed the degree of synchrony of 2D and 3D neuronal networks by computing the mean correlation coefficient (cc)^19^. We computed the cross-correlation matrix, 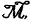, for all neuron pairs with entries *σ*_*CTij*_, varying between 0 and 1 (Methods) in 2D and 3D networks (Fig. 3k). We also computed the cross-correlation matrix, 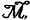, among the entire optical signals including their slow component. The entries of the matrix 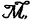 were *σ*_*SLOWij*_ and varied between −1 and 1 (Methods) in 2D and 3D conditions (Fig. 3l). In both cases, the cc was obtained by averaging all entries of the 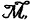 or 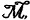 over all the experiments. The values of the cc for the calcium transients in 2D Glass and 2D G cultures were significantly lower (0.53 ± 0.006 and 0.59 ± 0.006, respectively) than that of the 3D-GF cultures (0.82 ± 0.005). We considered also the cross-correlation between the slow Ca^2+^ signals, which is a possibly more accurate measure of the degree of synchronization: the value of cc – obtained from 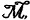-was lower for 2D Glass and 2D G cultures (0.33 ± 0.006 and 0.50 ± 0.007, respectively) than for 3D-GFs (0.62 ± 0.008) (Fig. 3m; ANOVA on ranks tested with Domm’s post-hoc test. n = 1497 couples of neurons for 6 neuronal cultures on 2D Glass; n = 1624 couples of neurons for 5 neuronal cultures on 2D G; n = 1093 couples of neurons for 6 neuronal cultures on 3D-GF). We examined also whether the value of cc depended on the distance between neurons: there was no difference when cc was computed among all active neurons in an image and for pairs of neurons separated by more than 200 μm (0.56 ± 0.009 for 2D Glass; 0.58 ± 0.008 for 2D G; 0.84 ± 0.007 for 3D-GF). These results show that the spontaneous firing of 3D networks is more synchronous than that of 2D networks and that the conductivity of graphene based materials, alone, does not affect the activity of neurons^36^,^37^ (Supplementary Information).

### Different degree of connectivity leads to different network regimes

After one week of culture, 3D networks exhibited two regimes: a highly synchronized (HS) regime, characterized by a very high synchrony of calcium transients (mean cc > 0.8), and a moderately synchronized (MS) regime, in which large synchronous transients coexisted with sparse smaller and uncorrelated transients (0.5 < mean cc < 0.8). The HS regime was very rarely observed in 2D networks grown on glass or 2D G obtained from the same batch of dissociated hippocampal neurons.

#### MS regime

**Figure 4:**
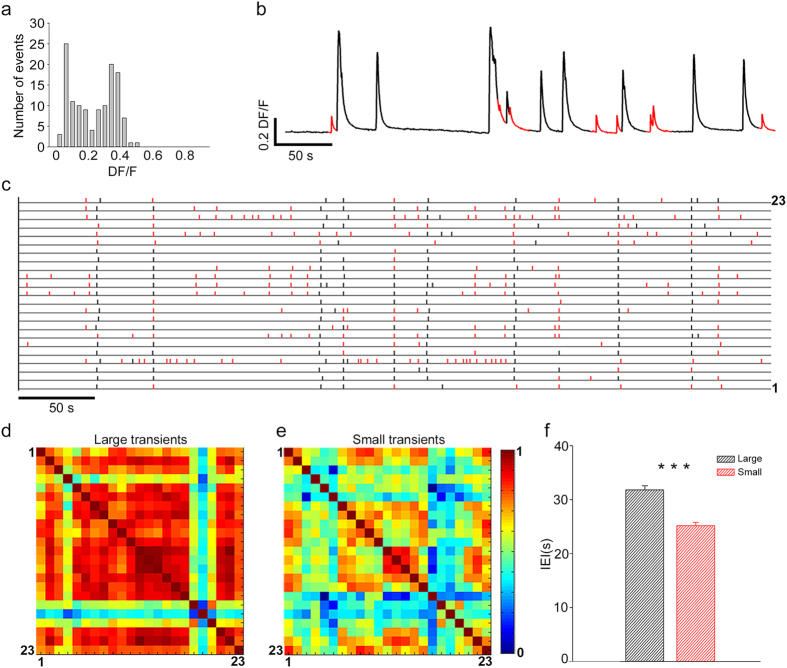
Small and large calcium transients. (**a**) Amplitude histograms of calcium transients obtained from one optical trace: two peaks are clearly present. (**b**) Representative optical trace with large (black) and small (red) calcium transients. (**c**) Raster plot of large (black dash) and small (red dash) transients for 23 different neurons. (**d,e**) Cross correlation matrices calculated for the large and small transients, respectively. (**f**) The mean value of IEI for large and small calcium transients; data from 3 experiments and a total of 50 neurons (***p < 0.001 Student t-test).

In several experiments, we observed from the cell body of the same neuron small and large calcium transients. Therefore, we computed the amplitude histograms of these transients during a period of up to 10–20 min before the occurrence of dye bleaching (Methods). For many neurons, these histograms had two well-separated peaks (Fig. 4a) that allowed the identification of two classes of calcium transients (Fig. 4b), i.e. small (red) and large (black). The former had approximately the same amplitude and could originate either from a single action potential (AP) or from a burst of a small number of APs, whereas the latter were likely due to a burst of several APs occurring in a window of 200–500 ms^38^. Visual inspection of raster plots of these transients (Fig. 4c) suggests that large calcium transients (black bars) were more synchronous than small calcium transients (red bars). Therefore, we analysed the degree of correlation of these two classes. The cross-correlation matrices 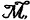 (Fig. 4d,e) show that the large calcium transients were more correlated (cc = 0.69 ± 0.010) than the smaller (cc = 0.45 ± 0.009; n = 518 couples of neurons. *Student*’*s t-test*). Therefore, in the MS regime large synchronous bursts of electrical activity coexisted with sparse firing which was poorly correlated. The frequency of the two types of signals differed significantly; the mean IEI of large synchronized transients was 31.7 ± 0.7 s, whereas that of small, poorly correlated transients was 25.1 ± 0.6 s (Fig. 4f. *Mann Whitney test*. n = 50 cells for 3 neuronal cultures).

#### HS regime

**Figure 5:**
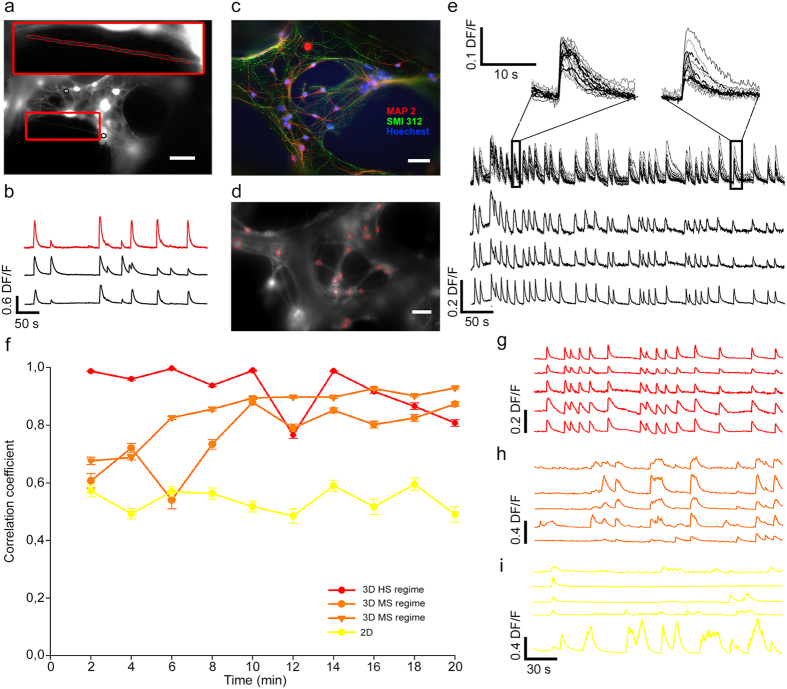
High connectivity leads to HS regime. (**a**) Fluorescent image of a neuronal culture grown on a 3D-GF loaded with Fluo-4 AM; a crossing neurite (inset) and two examples of ROIs (black circles) are shown. (**b**) Optical traces from the crossing neurite shown in a (red trace) and from two neighbouring neurons (black traces) obtained from the two ROIs indicated in a. (**c**) Fluorescence image of a neuronal culture grown on a 3D-GF where neurons cross and fill a pore of the scaffold with both neurites and cell bodies. Cells were stained for the somatodendritic neuronal marker MAP2 (red), axonal marker SMI 312 (green) and Hoechst 33342 nuclear stain (blue). (**d**) Example of a highly connected network as in c but loaded with Fluo-4 AM exhibiting the HS regime. (**e**) 24 superimposed optical traces (3 of them are shown separately in the bottom part) obtained from the 3D neuronal network shown in d; the rising phase of calcium transients is almost perfectly synchronized, as shown in the insets. (**f**) Time evolution of the correlation coefficient cc computed over a bin width of 2 minutes for 3D neuronal networks in the MS and HS regime and for 2D neuronal networks. (**g–i**) are examples of optical traces for each type of regime with the same colour code as in (**f**). Scale bar, 50 μm.

3D-GFs contained large pores with a diameter ranging from 100 to 500 μm. After 8–15 days of culture, we observed neurites able to cross the pores and bridge distances of 100–200 μm in the scaffold (Fig. 5a). Clear calcium transients that originated from crossing neurites were recorded (Fig. 5b red trace), and these transients were correlated with those that originated from neighbouring neurons (black traces). In some preparations, holes were not only filled with crossing neurites but also with the soma of neurons and glial cells, which appeared to be hanging in the pore (Fig. 5c).

3D-GFs with a high degree of connectivity, as indicated by the presence of the soma of neurons inside the holes and many crossing neurites (Fig. 5d), had calcium transients almost completely synchronous (Fig. 5e where 24 traces overlapped - top traces- and 3 isolated examples are reported - bottom traces). We defined this state as highly synchronous (HS) and the rising phase of these calcium transients matched perfectly (Fig. 5e, insets) within the limits of our time resolution (3–5 Hz). The falling phase of calcium transients from different neurons, however, had a different time course. This higher synchrony (cc = 0.93 ± 0.004; n = 483 couples of neurons) is attributed to a more extensive connectivity associated to the presence of crossings (or shortcuts) across the holes and the long neurites extending in 3D along the scaffold backbone.

#### Transitions between HS and MS

Neuronal cultures grown on 3D-GFs often exhibited clear transitions between the MS and HS regimes (Fig. 5f). During the 10–20 min of network activity, the value of cc – computed over a time window of 2–4 minutes - fluctuated between 0.8 and 1.0 (HS regime, Fig. 5f, red trace) as well as between 0.6 and 0.8 (MS regime, Fig. 5f, orange traces). A network remained in a given state for 2–5 minutes and frequently changed its degree of synchrony – i.e., the value of the cc. When cultures obtained from the same hippocampal tissue were grown on a 2D flat substrate – either glass or a graphene film – the value of cc remained confined between 0.4 and 0.6 (Fig. 5f, yellow trace). Only neuronal networks with a high number of crossings or shortcuts across holes, as those in Fig. 5c,d, remained in the HS state for the entire duration of the optical recording (Fig. 5e). In several 3D neuronal networks, transitions between the HS and the MS state were observed (orange symbols in Fig. 5f), and there were episodes in which calcium transients were highly synchronous (Fig. 5g) and in other episodes the degree of correlation was significantly lower (Fig. 5h). In the great majority of 2D neuronal networks the degree of synchrony was lower (yellow symbols in Fig. 5f,i). The transition from the MS to the HS regime is reminiscent of a phenomenon observed in 2D cultures after the addition of inhibitors of GABAergic pathways^39^.

#### Neuronal assemblies in synchrony change in time and space

**Figure 6:**
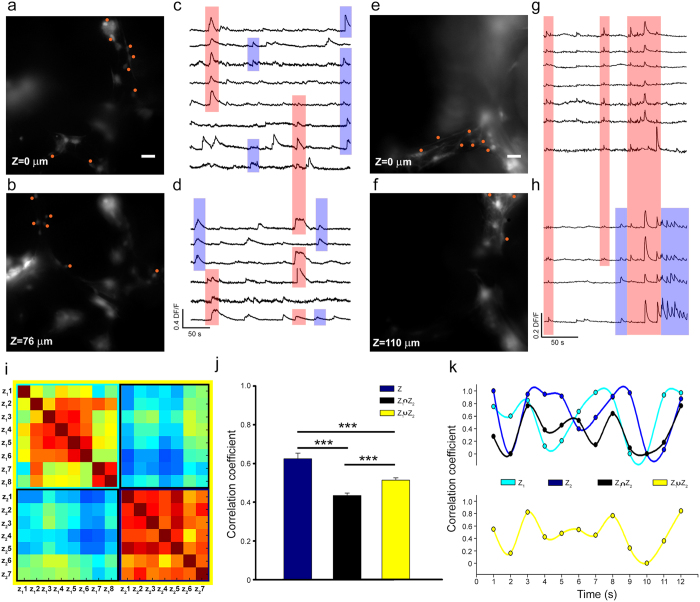
Assemblies of neurons firing in synchrony are dynamic and change both in space and time. (**a,b**,**e,f**) Fluorescent images of neuronal networks stained by Fluo-4-AM at two focal planes of the same 3D neuronal networks at different z heights (0–76 and 0–110 μm respectively). Calcium transients were obtained from identified neurons indicated by the red circles. (**c,d,g,h**) Traces obtained from neurons in (**a,b,e,f**) respectively. Calcium transients in synchrony on both planes are framed by pink shadow bars and those in synchrony only on a single plane by blue shadow bars. (**i**) Cross-correlation Matrix of calcium transients 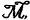 obtained at 2 different focal planes (z_1_ and z_2_). Data from 8 (7) neurons at the focal plane z_1_ (z_2_). 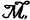 is partitioned in 4 regions, corresponding to the values of the cross-correlation among pairs of neurons in the same focal plane (the two squares along the diagonal, framed in light and dark blue) and among pairs of neurons in different focal planes (the two rectangles off the diagonal, framed in black). (**j**) The mean value of correlation coefficient for pairs of neurons on the same focal plane (blue), for pairs of neurons on different focal planes (black) and for all pairs of neurons (yellow). Collected data from 3 experiments (n = 79, 207 and 364 pairs of neurons, ***p < 0.001 ANOVA on ranks, Domm’s post-hoc test). (**k**) Time evolution of the correlation coefficient cc computed over a bin width of 60 s for pairs of neurons on the same focal plane (light blue and blue line), for pairs of neurons on different focal planes (black line) and for all pairs of neurons (yellow line). Scale bar, 50 μm.

3D-GFs have holes with a diameter of 100–500 μm, allowing visualization of neurons on focal planes separated by 100–200 μm along the z-axis (Supplementary Fig. S1). In this way we followed the spontaneous activity of 3D neuronal assemblies and we could observe possible changes in space and in time of the degree of synchrony. Indeed, by using a stage controlled by a fast piezoelectric device, we collected fluorescence images at focal planes separated in the axial (vertical) direction by 100–200 μm. Fluorescence images were obtained by acquiring 3 frames per second at each plane with a time shift of 167 ms (corresponding to 100 ms of exposure time and 67 ms for the movement of the stage). This delay between images collected at different focal planes is small in comparison of the duration of calcium transients (10–20 s) and represents the time resolution for the determination of synchrony. Calcium transients obtained from neurons with the soma on one plane (Fig. 6a,b,e,f; red circles) could be synchronous (Fig. 6c,d,g,h; blue shadow bars) and not in synchrony with calcium transients from neurons on focal planes at a distance of some tens of microns. Often, however, calcium transients obtained from focal planes at a distance of 76 (Fig. 6a–d) and 110 μm (Fig. 6e–h) were synchronous (pink shadow bars). These episodes of high synchrony in 3D were interspersed with periods of less correlated electrical activity (also Fig. 5f).

These results have two important consequences: firstly, they show that in 3D neuronal networks neurons extend neurites along the z-axis for several hundreds of microns so to form long-range connections; secondly, that the assemblies of neurons in synchrony are highly dynamic and change both in space and time, reminiscent of what is usually referred as frustrated synchronization in network theories^9^,^10^ and often observed in native 3D neuronal networks^19^.

We computed the cross-correlation matrix 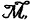 for all pairs of neurons positioned on both planes (Fig. 6i). Visual inspection of the matrix 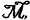, show a high degree of synchronization for pairs of neurons from the same focal plane (z_1_ or z_2_) (entries framed in light blue and dark blue in Fig. 6i). Pairs of neurons from different focal planes have a lower degree of synchrony (entries framed in black in Fig. 6i). Collected data from 3 neuronal cultures show that the mean value of cc was 0.61 ± 0.02 among calcium transients measured from neurons in the same focal plane. When pairs of neurons from different focal planes were considered the value of cc decreased to 0.43 ± 0.01 which was smaller than that of all pairs of neurons (cc = 0.51 ± 0.01). (Fig. 6j, ANOVA on ranks tested with Domm’s post-hoc test; 79, 207 and 364 pairs of neurons for z_1_ and z_2_, z_1_∩z_2_ and z_1_∪z_2_ respectively). These values of cc were obtained from averaging data over a time window of 20 minutes, but a different and more dynamic picture is observed when the cross-correlation matrix 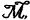 is computed on successive time windows of 60 s, as shown in Fig. 6k. The degree of synchrony of calcium transients among neurons on the same focal plane varies with time and when neurons on two different focal planes are considered there are episodes in which calcium transients are in phase or in anti-phase (Fig. 6k light and dark blue line). When pairs of neurons from different focal planes were considered, we observed episodes where calcium transients were highly synchronous (value of cc close to 0.8) and episodes of complete lack of synchrony (value of cc below 0.2). The overall degree of synchrony among all neurons varied significantly in time (Fig. 6k yellow line) and was slightly higher than that of pairs of neurons from distinct focal planes.

#### Maturation of 3D neuronal networks

**Figure 7:**
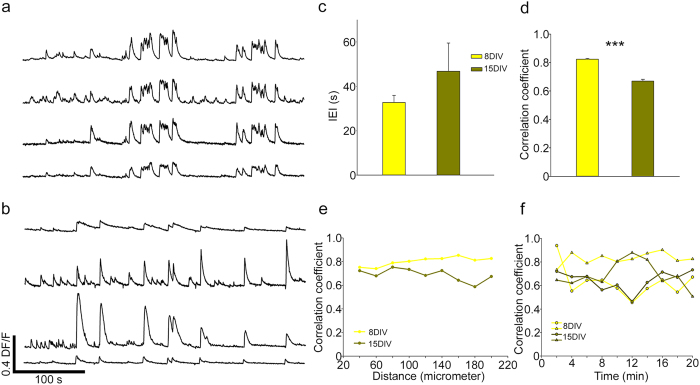
Changes of the degree of correlated activity of 3D neuronal networks during maturation. (**a,b**) Calcium transients of neurons cultured on 3D GF after 8DIV and 15 DIV respectively. (**c**) The mean value of IEI after 8DIV and 15DIV. (**d**) The correlation coefficient shows that there is a reduction in the network synchronization after 15DIV (***p < 0.001 Mann-Whitney test). (**e**) The correlation coefficient between pairs of neurons at different distances. While after 8DIV both near and far pairs of neurons are synchronous, at 15 DIV pairs of far neurons are less synchronous. (**f**) Time evolution of the correlation coefficient cc computed over a bin width of 2 minutes for 3D neuronal networks after 8DIV (yellow) and 15 DIV (green). The value of cc Correlation coefficient fluctuated similarly after 8 and 15DIV.

We investigated also changes of the spontaneous activity of 3D neuronal cultures during the maturation. We compared neuronal cultures obtained from the same batch of dissociated hippocampal neurons after one week (8–9 DIV) and two weeks (15–16 DIV) of culture. Calcium transients after one week of culture were rather synchronous (Fig. 7a) and the degree of synchrony decreased after two weeks (Fig. 7b). The mean IEI increased its value from 32.8 ± 3.0 to 46.8 ± 10.5 seconds (Fig. 7c). The value of the cc, instead, decreased from 0.82 ± 0.005 (8–9 DIV) to 0.53 ± 0.02 (15–16 DIV) in 3D-GFs and this change was statistically significant (Fig. 7d; *Mann-Whitney test*). The decreased correlation between calcium transients was more evident for pairs of neurons separated by a distance larger than 100 μm (Fig. 7e). The degree of synchrony, quantified by the value of cc, fluctuated similarly for 3D neuronal networks after one and two weeks of culture (Fig. 7f) and episodes of high synchrony (value of cc close to 0.9) were interspersed with periods of low synchrony (cc around 0.6). This reduction is very similar to what observed during maturation in the rat cortex^19^ where synchronization of calcium transients decreases at the second week of maturation. It is possible that the observed decrease of synchrony in our 3D neuronal cultures and in cortical networks have a similar origin associated to maturation.

## Discussion

The present manuscript demonstrates that the dynamics of 3D neuronal networks differ from those of 2D neuronal networks and better recapitulate what is observed *in vivo*^19^,^40^. This difference is due to a more extensive connectivity, which results in a more synchronous electrical activity^41^ and frustrated synchrony. Our results extend and complete previous recent investigations^42^,^43^, providing an experimental framework rationalizing theoretical results^9^,^10^,^23^,^24^,^25^ and explaining why 2D and 3D neuronal networks have different properties. In addition to this, we show that graphene scaffolds are a solid and biocompatible support useful in biomedical applications.

We have identified two main regimes of spontaneous activity that depend on the degree of 3D connectivity: a high connectivity leads to an almost complete synchronization of the bursting activity (HS regime); in the presence of a less extensive connectivity, synchronous bursts coexist with local uncorrelated firing (MS regime). A recent approach allowed to study simultaneously the calcium activity of multiple layers of the mouse cortex with cellular resolution^44^; acquisition at two different fields of view separated by almost 300 μm showed that some neurons can have a highly correlated activity. Our almost simultaneous measurement of calcium transients on two different focal planes of the 3D-GF, vertically separated by 70–150 μm, allowed us to observe correlated activity both between neurons of the same focal plane and between neurons from different planes. These results also show that the degree of correlated electrical activity is modular and changes in size with time, in agreement with network theory^9^,^10^,^23^,^24^,^25^ and experimental observations^19^,^40^. Therefore, neuronal networks grown on 3D-GFs recapitulate two basic properties of the complexity of the brain: firstly, the coexistence of local and global electrical activity (Fig. 4), and secondly, the existence of neuronal assembly with a degree of correlated electrical activity varying in space and time (Figs 1, 5 and 6). These two properties are not shared by 2D neuronal networks, and are the consequence of the dimensionality of networks grown on 3D-GFs.

Glial cells grown on 3D scaffolds maintain the *in vivo*-like complex morphology^2^,^45^. Almost 80% of astrocytes extended processes on our 3D-GFs, whereas glia with processes represented less than 40% of GFAP-positive cells on glass. The amount of “undifferentiated” astrocytes (i.e., lacking processes) reached almost 80% on 2D G, the opposite of what we observed on 3D-GFs. Therefore, the 3D topology, rather than graphene itself, promotes the extension of glial processes in all three dimensions, as it does *in vivo*^2^,^46^.

The diameter of pores in 3D hydrogel scaffolds^47^ and 3D nanofibers^2^ are on the order of some tens of micrometres, varying from 20 to 60 μm. However, the pores in our 3D-GF scaffolds are larger by almost 1 order of magnitude. As shown in Fig. 5, neurites and even the cell bodies of neurons and glial cells could be found inside these holes. Crossings more often occurred near the edges of the holes, where the distance is shorter and more anchoring sites are present. Large pores lead to enhanced nutrient and oxygen diffusion^48^ and the optimization of pore size is crucial.

## Materials and Methods

### Construction of the simulated 3D network and Kuramoto model

In order to construct the 3D network, first we place the nodes (neurons) along a 3D Pythagoras fractal tree.

This tree is formed by n = 8 number of iterations, it has a vector scale factor r = [0.5,0.8,0.8] a vector of azimuth angles of the fractal generator phi = [0,2*pi/3,4*pi/3], a vector of polar angles in fractal generator relative to the trunk chi = [pi/3,pi/3,pi/3], and coordinate of the trunk xb, yb and zb given by [0,0],[0,0],[0,1]. This tree can be generated with the FraktalT3D MATLAB code using the command

FraktalT3D (n,[0.5,0.8,0.8],[0,2*pi/3,4*pi/3],[pi/3,pi/3,pi/3],[0,0],[0,0],[0,1]).

Along the branches of the tree we place randomly the nodes (neurons) of the simulated network with an average density ρ = 3 neurons/unit length. We indicate with *N* the number of such neurons.

The simulated neuronal network is generated by placing short-range links between the neurons *i* and *j* with probability


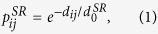


and long-range links with probability


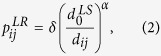


where d_ij_ is the 3D distance between neuron *i* and neuron *j*, and 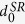, 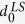, δ and *α* are parameters determining the topology of the network.

For a wide range of parameter values the networks generated in this way are small world, have a modular structure and the distribution of the nodes is fractal.

On these networks we simulated a Kuramoto model of coupled oscillators, by numerically integrating the equations


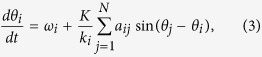


for *i = 1*,*2… N*, where θ_i_ is the phase of the oscillator *i*, *K* is the parameter determining the intensity of the coupling between the oscillators, ω_i_ is the intrinsic frequency of the oscillator *i* and is drawn randomly from a Gaussian distribution with zero average and unitary standard deviation, *a*_*ij*_ is the adjacency matrix of the network indication which neurons are connected together, and *k*_*i*_ is the degree of node *i*, i.e.


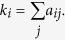


In order to evaluate the synchronization property of the 3D network we have measured the order parameter *R* given by


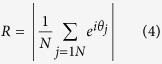


which takes values between 0 and 1.

Large values of *R* indicate phase synchronizations while *R* ≈ *0* indicates absence of synchronization.

We found, that for networks which are at the same time small world and fractal, three different phases of the synchronization dynamics occur as a function of the coupling *K*. These are: for high values of *K*, the fully synchronized phase characterized by large values of R which reach a steady state in time, for intermediate values of *K*, the frustrated synchronization characterized by intermediate values of *R* which do not reach a steady state in time, and for low values of *K*, the absence of synchronization characterized by very small values of *R*. These phases as described in the main text and typical simulations results are shown in Fig. 1e.

We note here that the network considered in Fig. 1d,e it is constructed by taking 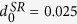, 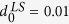, *α* = 2.5 and a dynamics given by equation (3) with *K* = *1, K = 9* and *K* = *25*.

### Scaffold preparation

Graphene samples were synthesized using the chemical vapour deposition (CVD) method as described previously^14^,^49^,^50^. Briefly, the 3D-GFs were made via CVD using Ni foam as a template, whereas the 2D graphene films were prepared using a Cu foil as substrate. All heavy metal components were then chemically removed, and the substrates were rinsed with HNO_3_, HCl and running water for at least 72 h to remove the etching agents. For sterilization, the scaffolds were treated with UV light for 20 min, followed by decreasing concentrations of ethanol (100%, 75%, 50% for 10 min). Finally, the scaffolds were rinsed with sterile deionized water (twice for 10 min).

### Neuronal preparation and culture

Hippocampal neurons from Wistar rats (P2-P3) were prepared in accordance with the guidelines of the Italian Animal Welfare Act, and their use was approved by the Local Veterinary Service, the SISSA Ethics Committee board and the National Ministry of Health (Permit Number: 630-III/14) in accordance with the European Union guidelines for animal care (d.1.116/92; 86/609/C.E.). The animals were anaesthetized with CO_2_ and sacrificed by decapitation, and all efforts were made to minimize suffering. All substrates (2D glass coverslips, 2D graphene films and 3D-GFs) were coated with 50 μg/ml poly-L-ornithine (Sigma-Aldrich, St. Louis, MO, USA) overnight, soaked in culture medium overnight and coated with Matrigel just before cells seeding (Corning, Tewksbury MA, USA). Dissociated cells were plated at a concentration of 6 × 10^5^ cells/ml on 2D substrates and 2.4 × 10^6^ cells/ml on 3D-GF in minimum essential medium (MEM) with GlutaMAX^TM^ supplemented with 10% foetal bovine serum (FBS, all from Invitrogen, Life Technologies, Gaithersburg, MD, USA), 0.6% D-glucose, 15 mM Hepes, 0.1 mg/ml apo-transferrin, 30 μg/ml insulin, 0.1 μg/ml D-biotin, 1 μM vitamin B12 (all from Sigma-Aldrich), and 2.5 μg/ml gentamycin (Life Technologies). After 48 hours, 2 μM cytosine-β*-*D-arabinofuranoside (Ara-C; Sigma-Aldrich) was added to the culture medium to block glial cell proliferation, and the concentration of FBS was decreased to 5%. Half of the medium was changed every 2–3 days. The neuronal cultures were maintained in an incubator at 37 °C, 5% CO_2_ and 95% relative humidity. The cell concentration was adjusted to ensure comparable cell numbers on all substrates. Unlike the 2D substrates, on which all plated cells uniformly deposit on the surface, the 3D-GFs retain cells, which permeate the pores.

### Calcium Imaging

The cells were loaded with a cell-permeable calcium dye Fluo4-AM (Life Technologies) by incubating them with 4 μM Fluo4-AM (dissolved in anhydrous DMSO (Sigma-Aldrich), stock solution 4 mM) and Pluronic F-127 20% solution in DMSO (Life Technologies) at a ratio of 1:1 in Ringer’s solution (145 mM NaCl, 3 mM KCl, 1.5 mM CaCl_2_, 1 mM MgCl_2_, 10 mM glucose and 10 mM Hepes, pH 7.4) at 37 °C for 1 hour. After incubation, the cultures were washed and then transferred to the stage of a Nikon Eclipse Ti-U inverted microscope equipped with a piezoelectric table (Nano-ZI Series 500 μm range, Mad City Labs), an HBO 103 W/2 mercury short arc lamp (Osram, Munich, Germany), a mirror unit (exciter filter BP 465–495 nm, dichroic 505 nm, emission filter BP 515–555) and an Electron Multiplier CCD Camera C9100-13 (Hamamatsu Photonics, Japan). The experiments were performed at RT, and images were acquired using the NIS Element software (Nikon, Japan) with an S-Fluor 20x/0.75 NA objective at a sampling rate of 3–10 Hz with a spatial resolution of 256 × 256 pixels for 10–20 min. To avoid saturation of the signals, excitation light intensity was attenuated by ND4 and ND8 neutral density filters (Nikon).

### Data Analysis

#### Ca^2+^ imaging processing and analysis

The initial video was processed with the ImageJ (U. S. National Institutes of Health, Bethesda, MA) software. The image sequences were then analysed as described previously^51^. Briefly, neurons were localized, and an appropriate region of interest (ROI) was selected to subtract the background. Appropriate ROIs around the cells bodies were then selected. The time course of the fluorescence intensity, I_f_(t), in this ROI was displayed, and any decay, which is a consequence of dye bleaching, was evaluated. The Ca^2+^ transients of each cell signal were extracted in a semi-automatic manner by selecting a threshold for the smallest detectable peak that was equal to three times the standard deviation of the baseline. Subsequently, the decay of I_f_(t) was fitted to a cubic spline interpolating I_f_(t) at 10 or 20 points. I_f_(t) was then fitted to the original optical signal to compensate for dye bleaching, and the fractional optical signal was calculated as follows: DF/F = (Y(t)+I_f_(t))/I_f_(0), where I_f_(0) is the fluorescence intensity at the beginning of the recording.

#### Computation of raster plot and correlation coefficient of Ca^2+^ transient occurrence

The times, *t*_*i*_, at which transient peaks occurred are presented in a conventional raster plot. To isolate the smaller transients from the larger ones, the single traces were considered independently. The amplitude distribution of peaks was calculated to separate the two different classes of events. Based on this distribution, a threshold was set to approximately 30% of the maximum amplitude. All peaks under the threshold were considered small, whereas all other peaks were considered to be large calcium transients.

The correlation coefficient of the calcium transients for neuron *i* and neuron *j* (*σ*_*CTij*_) was computed as follows: The total recording time, *T*_*tot*_, was divided into *N* intervals (1,..,n,…,N) of a duration *Δt*. Thus, if *f*_*in*_ and *f*_*jn*_ are the number of calcium transients of neuron *i* and neuron *j* in the time interval *Δt*_*n*_, then


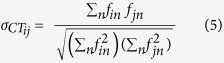


such that *σ*_*CTij*_ depends on *Δt* and varies between 0 and 1. The range of explored values of *Δt* was 20 s.

#### Computation of cross-correlation of slow Ca^2+^ oscillation

Because we observed that Ca^2+^ transients can occur both during a positive phase and a negative phase of Ca^2+^ fluctuation, we also analysed and computed the correlation coefficient of slow Ca^2+^ oscillation. The correlation coefficient of this type of oscillation can be negative, whereas *σ*_*CTij*_ can only vary between 0 and 1. The correlation coefficient of slow Ca^2+^ oscillation obtained for neuron *i* and neuron *j* (*σ*_*SLOWij*_) was computed as follows:

If *s*_*in*_ is the slow signal from neuron *i* at time *t*_*n*_, its mean value, 〈*s*_*i*_〉, is given by 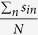 where *N* is the total number of available samples.


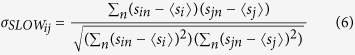


so that *σ*_*SLOWij*_ varies between −1 and 1 and *σ*_*SLOWij*_ was computed at the same time interval of *σ*_*CTij*_.

### Morphological and immunocytochemical analysis

Cells were fixed in 4% paraformaldehyde containing 0.15% picric acid in phosphate-buffered saline (PBS), saturated with 0.1 M glycine, permeabilized with 0.1% Triton X-100, saturated with 0.5% BSA (all from Sigma-Aldrich) in PBS and then incubated for 1 h with primary antibodies: mouse monoclonal glial fibrillary acidic protein (GFAP), rabbit polyclonal against MAP2 and GABA (all from Sigma-Aldrich), anti-β-tubulin III (TUJ1) and SMI 312 mouse monoclonal antibodies (Covance, Berkeley, CA). The secondary antibodies were goat anti-rabbit Alexa Fluor^®^ 488, goat anti-mouse Alexa Fluor^®^ 594, goat anti-mouse immunoglobulin (Ig) G_1_ Alexa Fluor^®^ 488, goat anti-mouse IgG_2a_ Alexa Fluor^®^ 594, (all from Life Technologies) and the incubation time was 30 min. Nuclei were stained with 2 μg/ml in PBS Hoechst 33342 (Sigma-Aldrich) for 5 min. All the incubations were performed at room temperature (20–22 °C). The cells were examined using a Leica DM6000 fluorescent microscope equipped with DIC and fluorescence optics, CCD camera and Volocity 5.4 3D imaging software (PerkinElmer, Coventry, UK). The fluorescence images were collected with a 20x magnification and 0.5 NA objective. For each image at least 30 slices were acquired with slice spacing of 0.5 μm. Image J by W. Rasband (developed at the U.S. National Institutes of Health and available at http://rsbweb.nih.gov/ij/) was used for image processing.

### Statistical analysis

Data are shown as the mean ± s.e.m from at least three neuronal cultures. For the morphological analysis of immunofluorescence images (Fig. 2), n refers to the number of images analysed, and the number in brackets refers to total number of cells analysed. The quantified activity (IEI and Cross-correlation) and morphological data were analysed with the ANOVA test followed by post hoc comparisons using the software SygmaPlot 10.0. Differences among two groups were evaluated with Kolmogorov-Smirnov test, Student’s-t test or Mann-Whitney test (Statistica 6.0 – StatSoft Italy). The number of replicates and statistical tests used for each experiment are mentioned in the respective figure legends or in the Results. Significance was set to *p < 0.05, **p < 0.01 and ***p < 0.001.

## Additional Information

**How to cite this article**: Ulloa Severino, F. P. *et al*. The role of dimensionality in neuronal network dynamics. *Sci. Rep.* **6**, 29640; doi: 10.1038/srep29640 (2016).

## Supplementary Material

Supplementary Information

Supplementary Video S1

Supplementary Video S2
