# Supplementary material for: The role of dimensionality in neuronal network dynamics
Source: Sci Rep. 2016 Jul 11;6:29640. doi: 10.1038/srep29640 (PMC4939604; doi:10.1038/srep29640)
Supplement: Supplementary Information [file srep29640-s1.pdf]

Supplementary Information

The role of dimensionality in neuronal network dynamics

Francesco Paolo Ulloa Severino[1†], Jelena Ban[1†], Qin Song[2], Mingliang Tang[3], Ginestra Bianconi[4], Guosheng Cheng[2*] and Vincent Torre[1*]

[1]Neurobiology Sector, International School for Advanced Studies (SISSA), via Bonomea, 265, 34136 Trieste, Italy

[2]Key Laboratory of Nano-Bio Interface, Suzhou Institute of Nano-tech and Nano-bionics, Chinese Academy of Sciences, 398 Ruoshui Road, Jiangsu 215123, China

[3]Institute of Life Sciences, Southeast University, Sipailou 2, Nanjing 210096, China

[4]School of Mathematical Sciences, Queen Mary University of London, Mile End Rd, London E1 4NS, United Kingdom

†These authors contributed equally

* Corresponding authors:

Vincent Torre, email: torre@sissa.it

Cheng Guosheng, email: gscheng2006@sinano.ac.cn

Supplementary Figure S1

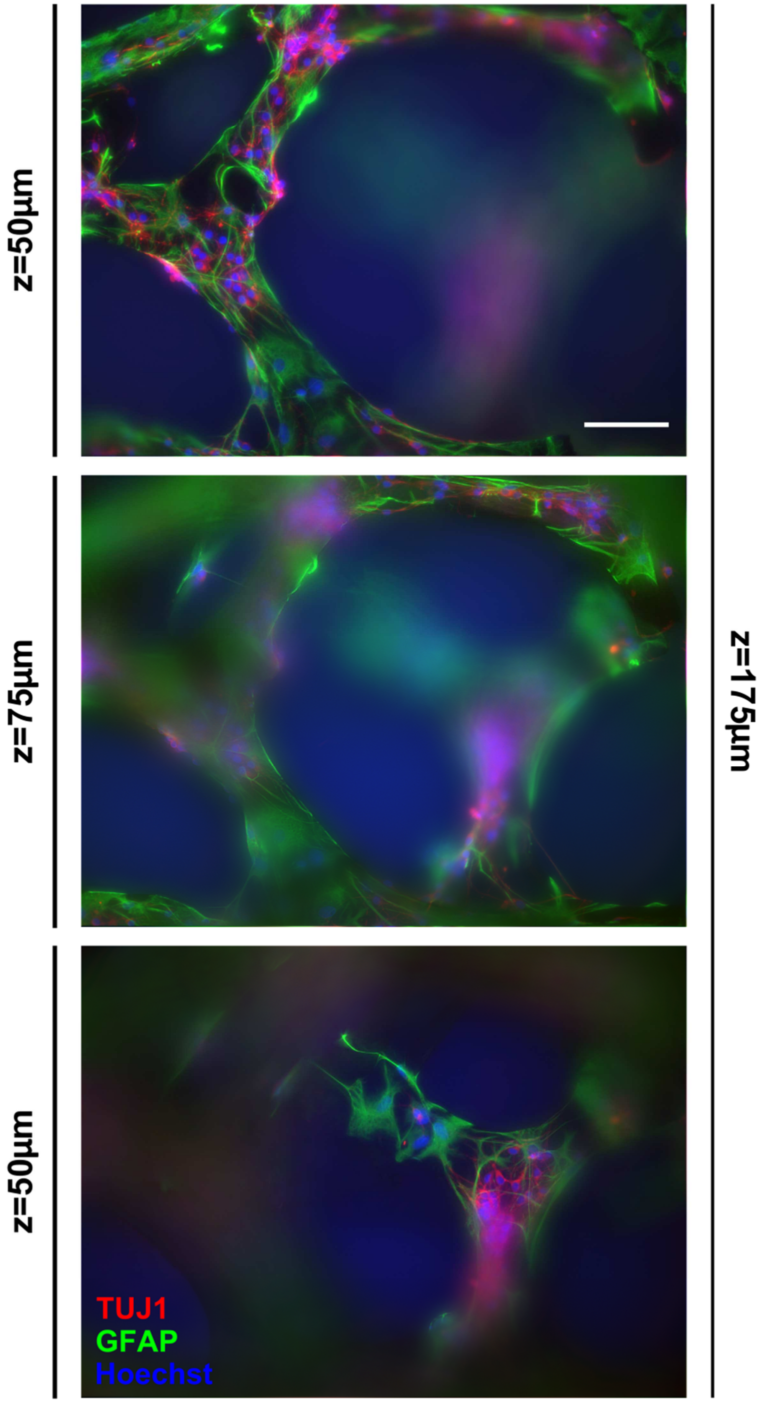

Supplementary Figure S1. Neuronal network is continuous throughout the 3D-GF backbone. From top to bottom: three consecutive z-projections for the 175 µm thick z-stack. The thickness of each sub-stack is indicated on the left and the total z-stack thickness is indicated on the right. Images were acquired with slice spacing of 0.5 µm and derive from the neuronal culture grown for 7 DIV on 3D-GFs and stained for β-tubulin III (TUJ1, red), glial fibrillary acidic protein (GFAP, green) and Hoechst 33342 nuclear stain (blue). Scale bar, 100 µm.

Supplementary Figure S2

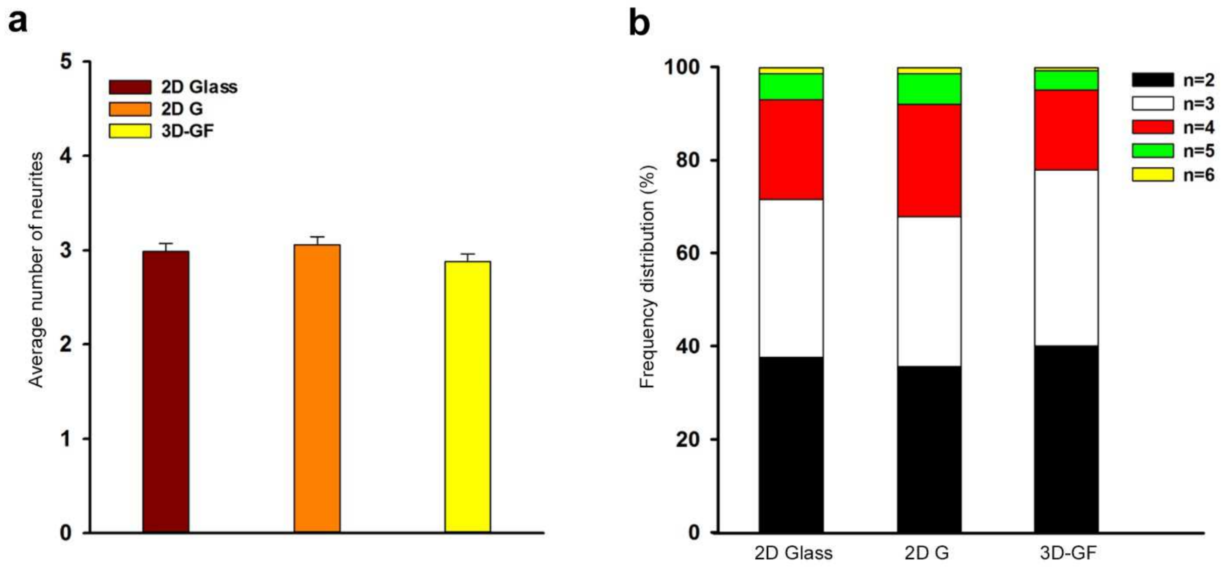

Supplementary Figure S2. Neurite outgrowth is similar between 2D and 3D neuronal networks. (a) The average number of neurite per neuron was obtained from fluorescence images of neurons stained for MAP2 at 7 DIV. (b) Frequency distribution of neurite numbers for glass, 2D G and 3D-GFs. n refers to number of neurites per neuron.

Supplementary Figure S3

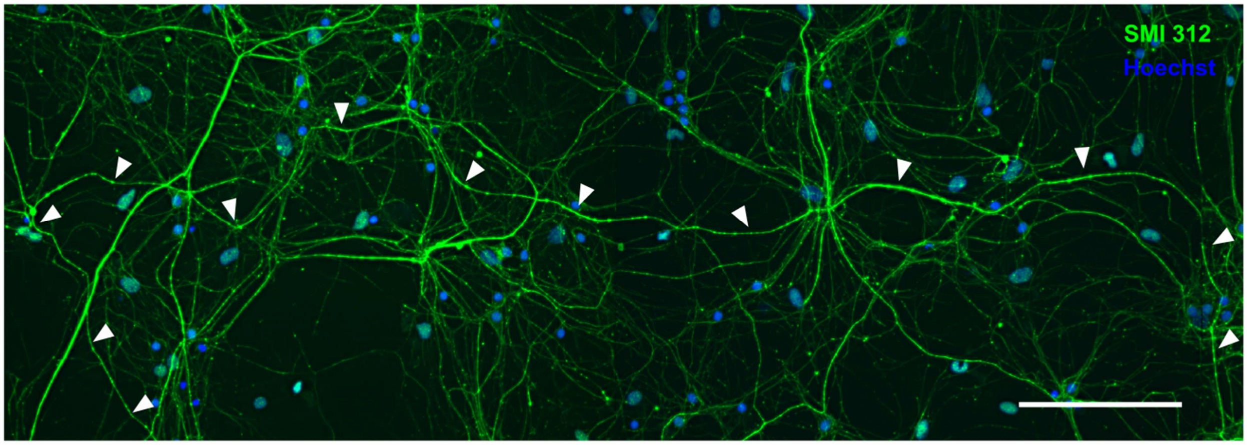

Supplementary Figure S3. After two weeks of culture axons can reach millimeter length. Fluorescent image of hippocampal culture at 14 DIV cultured on glass and stained for axonal marker SMI 312. Cell nuclei were marked by Hoechst 33342. Arrowheads follow the axon that extends for several hundreds of micrometer reaching millimeter length. Similar axon outgrowth was observed also on 2D G and 3D-GFs. Scale bar, 100 µm.

Supplementary Figure S4

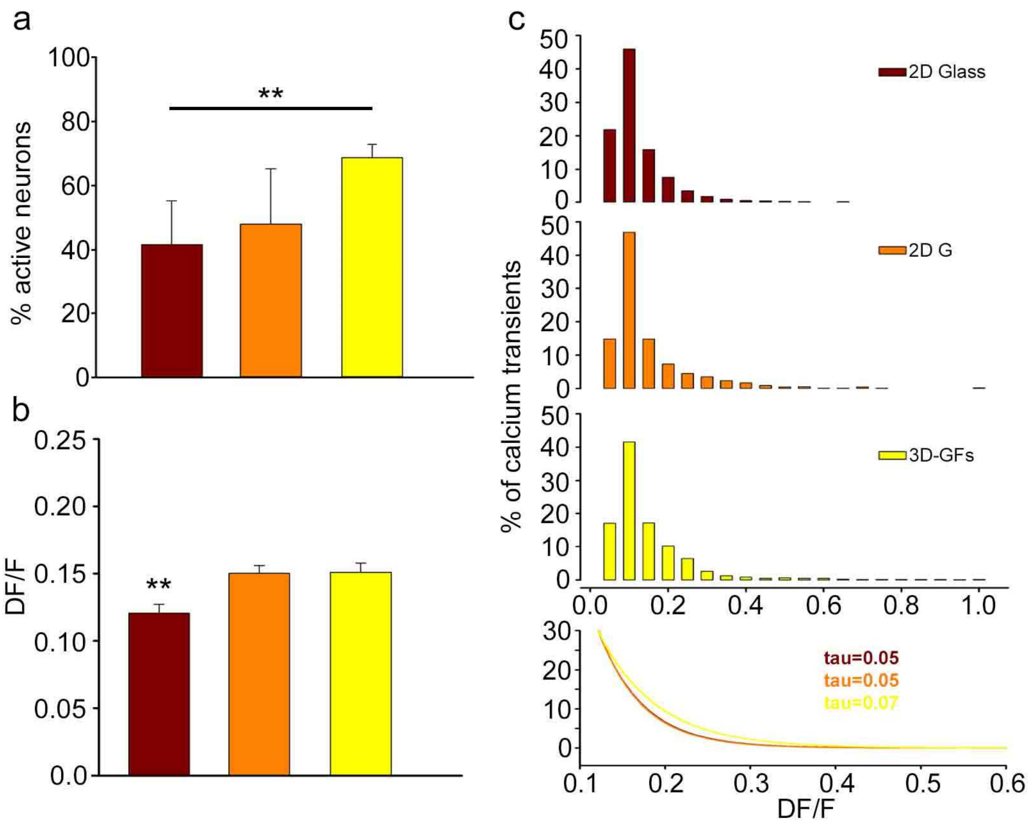

Supplementary Figure S4. Standard analysis of network activity. (a) Percentage of active neurons for 2D Glass, 2D G and 3D-GFs respectively. Significant difference is reported comparing 2D Glass and 3D-GFs (**=$p<0.01$ ANOVA on ranks, Domm's post-hoc test). (b) Weighted mean of Ca^{2+}-transients amplitude (averaged over the single neurons). 2D G and 3D-GFs have comparable values, neurons on 2D Glass show a significant reduction (**=$p<0.01$ ANOVA on ranks, Domm's post-hoc test). (c) Frequency distribution of the amplitudes for the three conditions. The bottom panel show the exponential fitting curve used to evaluate the decay time of the chart.

Supplementary Information 1

Comparison of the 2D cultures

In this work we used two different experimental controls: glass coverslip (2D Glass) and 2D graphene films (2D G). Standard 2D neuronal networks are most commonly cultured on glass, while graphene films (see Methods) represent an additional control for material. In order to understand if the differences observed with neuronal networks on 3D scaffolds are due to graphene or 3D arrangement, we performed additional morphological and functional analysis in which we compared 2D cultures.

Supplementary Figure S5 shows that after 7 DIV - although glia to neuron ratio is similar between 2D cultures (see Figure 2 of the manuscript) - astrocytes on 2D G are larger, have less processes and cover the substrate completely (see Supplementary Fig. S5b). These differences were even more pronounced after 14 DIV where cell-free regions on glass surface increased while on 2D G astrocytes retained their morphology and completely covered the surface (compare panel c and d of Supplementary Fig. S5). Similarly, after 14 DIV we observe a denser neuronal network on 2D G, as shown by the staining of axons with SMI 312 (Supplementary Figure S5e-f). All these observations indicate that on 2D G cells survive better than on glass.

The observed differences could be related to the roughness of the graphene films surface that could enhance cell adhesion[1]. Further investigations are required using adhesion markers such as integrin, paxillin or vinculin.

Morphological observations can be confirmed by the functional data. We observed that graphene itself does not affect the rate of activity (see Figure 3j), but higher density of neurites could have some effect on the synchronization (see Figure 3m). However, it still remains significantly different (i.e. lower) to what observed for 3D cultures.

Moreover, it has been reported that the electrical conductivity of the materials alone, as well as their nanostructure, did not affect the excitability of neurons suggesting the synergy of multiple properties as a key factor[2]. In addition, few-layer graphene does not affect the frequency of the neuronal activity[3].

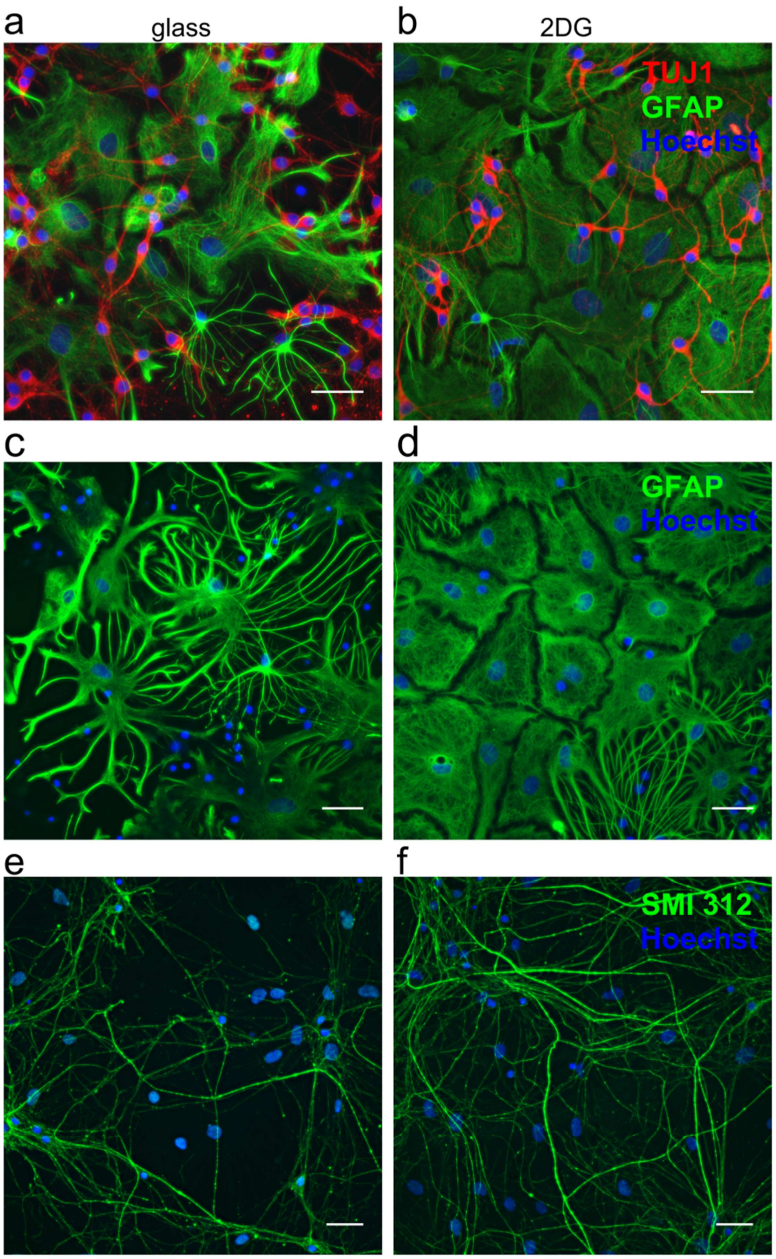

Supplementary Figure S5. Morphological comparison of 2D cultures. Hippocampal culture at 7 DIV on glass (a) and 2D G (b) stained for β-tubulin III (TUJ1, red) and glial fibrillary acidic protein (GFAP, green). (c-d) GFAP-positive astrocytes (green) and (e-f) SMI 312-positive axons (green) after 14 DIV on glass (left) and 2D G (right) respectively. Nuclei were stained with Hoechst 33342 nuclear stain (blue). Scale bar, 50µm.

1. Lorenzoni, M., Brandi, F., Dante, S., Giugni, A. & Torre, B. Simple and effective graphene laser processing for neuron patterning application. *Sci. Rep.* **3,** 1954 (2013).
2. Cellot, G. *et al.* Carbon nanotubes might improve neuronal performance by favouring electrical shortcuts. *Nat Nano* **4,** 126–133 (2009).
3. Fabbro, A. *et al.* Graphene-Based Interfaces Do Not Alter Target Nerve Cells. *ACS Nano* **10,** 615–623 (2016).

Supplementary Videos

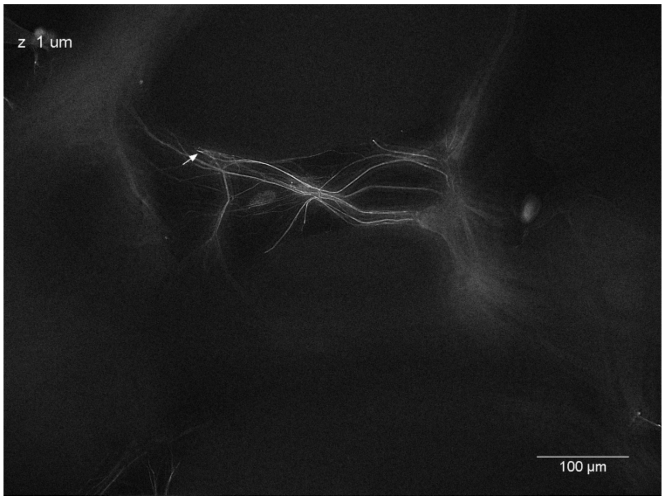

Supplementary Video S1. Axonal projection in 3D space. Axons on 3D-GFs project in all three dimensions connecting the different layers and extending for several hundreds of micrometers. Images were acquired with 20x and 0,5 NA objective for a total thickness of 95 µm with slice spacing of 1 µm. Cells were cultured for 2 weeks on 3D-GFs and stained with axonal marker SMI 312. The arrow follows the axon that connects different planes of the scaffold.

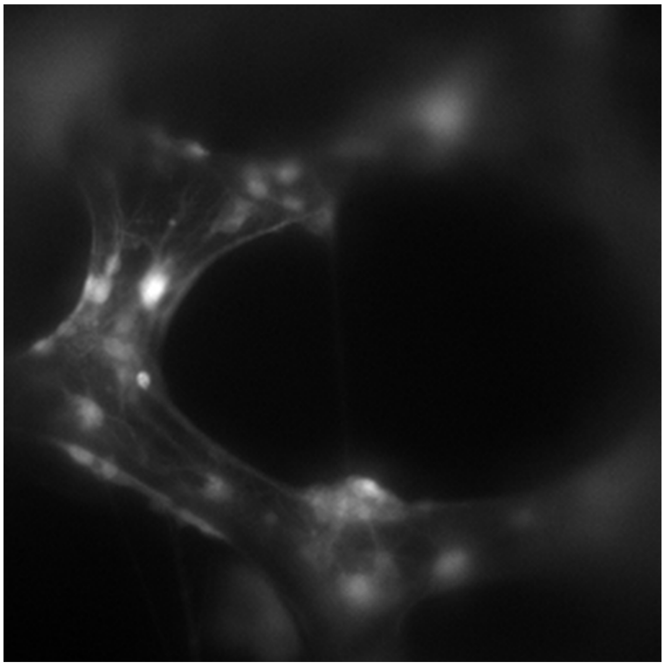

Supplementary Video S2. Calcium imaging. Neuronal culture loaded with 4µM Fluo-4-AM calcium indicator on 3D-GF. Images were acquired with 20x and 0,75 NA objective, 5 Hz, a spatial resolution of 256 × 256 pixels and for 10 min. The movie reproduction has been made using 25 frames per second.
